# Reliability and validity of eye–hand coordination pointing tests for older adults

**DOI:** 10.1097/MD.0000000000036025

**Published:** 2023-11-17

**Authors:** Kazuo Saito, Makoto Suzuki, Maki Koyama, Junichi Yamamoto, Toshiko Futaki

**Affiliations:** a Faculty of Health Sciences, Tokyo Kasei University, Saitama, Japan; b Faculty of Systems Design, Tokyo Metropolitan University, Tokyo, Japan; c River Basin Research Center, Gifu University, Gifu, Japan; d Faculty of Healthy Sciences, Okayama Healthcare Professional University, Okayama, Japan.

**Keywords:** cognitive functions, eye–hand coordination, motor function, older adults, rhythmic movement

## Abstract

To examine the reliability and validity of eye–hand coordination pointing with pencil test (EHCPPT), which evaluates the spatial and temporal displacement of rhythmic movements. One hundred and thirty-five older adults participated in our study. Reproducibility of the EHCPPT was assessed by the participants tapping on the center of the circles using a tablet pen in response to 2 types of stimulus sound sequences (Test A and B) across 2 separate trials, the first and second half. Construct validity was assessed by comparison in the distances between ultimate and current processing abilities, based on the spatial and temporal displacement relationship across Test A and Test B. Concurrent validity was assessed by examining the relationship between the distances between ultimate and current processing abilities and the motor and cognitive functions. Spatial and temporal displacements showed an excellent intraclass correlation coefficient in both Tests A and B of EHCPPT. The distance between ultimate and current processing ability, based on the relationship of spatial and temporal displacement, was significantly shorter in Test A compared to Test B. Spatial and temporal displacements were correlated with motor and cognitive functions. The spatial and temporal displacements of EHCPPT indicated reproducibility and validity in older adults. The EHCPPT may serve as a rhythmic movement reflecting motor and cognitive functions.

## 1. Introduction

Rhythmic movements, such as brushing teeth, using a kitchen knife, or operating a keyboard, are crucial in daily life. These movements necessitate the ongoing processing of spatial and temporal information to correct motion errors in subsequent cycles.^[1]^ The ability to process spatial and temporal information becomes apparent in the trade-off between accuracy and speed. This trade-off is a fundamental property of the kinematics associated with neuromuscular networks.^[2]^ Recently, this trade-off has been employed to assess and characterize the performance of various input systems in human-computer interaction.^[3,4]^ Moreover, previous studies have observed that rhythmic movements relying on spatial and temporal information necessitate not only motor but also cognitive function.^[1]^ There is a correlation between upper limb motor control, attention function, and short-term memory.^[5]^ Prior research also indicates that tasks involving rhythmic movements require substantial motor and cognitive resources.^[5]^ These findings suggest that rhythmic movements, predicated on spatial and temporal information, are related to both upper and lower extremity motor functions and cognitive functions.

In this context, various rhythmic movement tasks such as upper extremity coordination and finger tapping have been used to assess the cognitive and motor functions.^[1,5–7]^ Specifically, the finger tapping task^[6,7]^ may be widely used to quantify rhythmic movement function and may offer several advantages such as time efficiency, low cost, free weights, and ease of scoring. Additionally, the finger tapping task has been used for neurophysiological studies and for the clinical evaluation of patients with Parkinson’s^[8]^ or cerebrovascular disease.^[9,10]^ These tasks are highly sensitive to coordinated movements, and hence serve as an evaluation index for physical and cognitive functions. However, rhythmic movement tasks like the finger tapping task are unable to assess the processing ability of spatial and temporal information related to the trade-off between accuracy and speed. Consequently, we developed a rhythmic movement test, which includes finger tapping, and we have named it the Eye–Hand Coordination Pointing with Pencil Test (EHCPPT).^[11]^ The EHCPPT can objectively evaluate the spatial and temporal displacement occurring during rhythmic performance. In a preliminary study, we observed that the EHCPPT was capable of capturing the characteristics of illnesses related to motor functions.^[11]^

Comprehensive assessment including motor and cognitive functions in daily life has been recommended by the geriatric society.^[1]^ However, a comprehensive assessment for patients and older individuals requires specialized knowledge and evaluation time. If an EHCPPT could assess the rhythmic movements based on accuracy and speed trade-off reflecting various motor and cognitive functions of older individuals and patients with various impairments, their motor and cognitive function could be comprehensively and objectively assessed, and training for motor and cognitive dysfunction could be more evidence-based. According to evidence on rhythmic movement tasks,^[1,5,6]^ we hypothesized that the EHCPPT would consistently assess rhythmic movement function based on accuracy and speed trade-off of healthy adults and patients with various impairments. Therefore, the purpose of this study was to investigate reproducibility and validity of an EHCPPT to evaluate the spatial and temporal displacement of rhythmic movements.

## 2. Methods

### 2.1. EHCPPT

**Figure 1.**
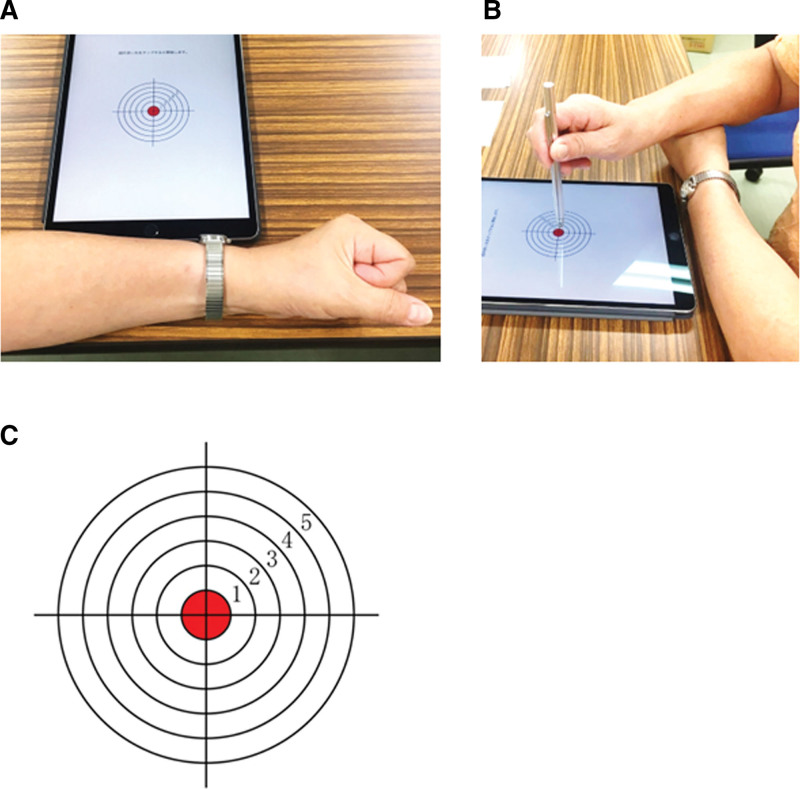
The HCPPT. Participants are seated in a chair, with their non-dominant hand resting on the desk (A) and their dominant hand placed over it (B). Participants are instructed to tap the center of circles using a tablet pen in response to 2 distinct types of stimulus sound sequences (C). EHCPPT = Eye–Hand Coordination Pointing with Pencil Test.

The participants were seated on a chair or wheelchair, with their torso away from the desk by approximately one fist, with their non-dominant hand on the desk (Fig. 1A) and the dominant hand on top of it (Fig. 1B). The test was presented using a pointing-task app (EHCPPT) on an Apple iPad (MP2F2J/A) screen. Using a special pen in their dominant hand, the participants tapped on the center of 6 concentric circles on the app screen in response to stimulus sounds (i.e., sounds used for rhythmic movement tests) produced at regular intervals for 2 EHCPPT (Fig. 1C). The test works in the following manner. In Test A, the participants tapped on the target immediately after every low-pitched beep (1 kHz; 60 beeps) produced at 1-second intervals. In Test B, 65 low-pitched (1 kHz) and 35 high-pitched (2 kHz) beeps were played randomly, and the participants were expected to tap only in response to the high-pitched beep. The app captured the XY coordinates (in pixels) on the iPad screen when the participants’ pen touched the screen. Subsequently, the app converted the coordinate into an XY coordinate (in cm), with the center of the concentric circles on the screen (the target center) as the origin, and stored the value along with the touch time.

**Figure 2.**
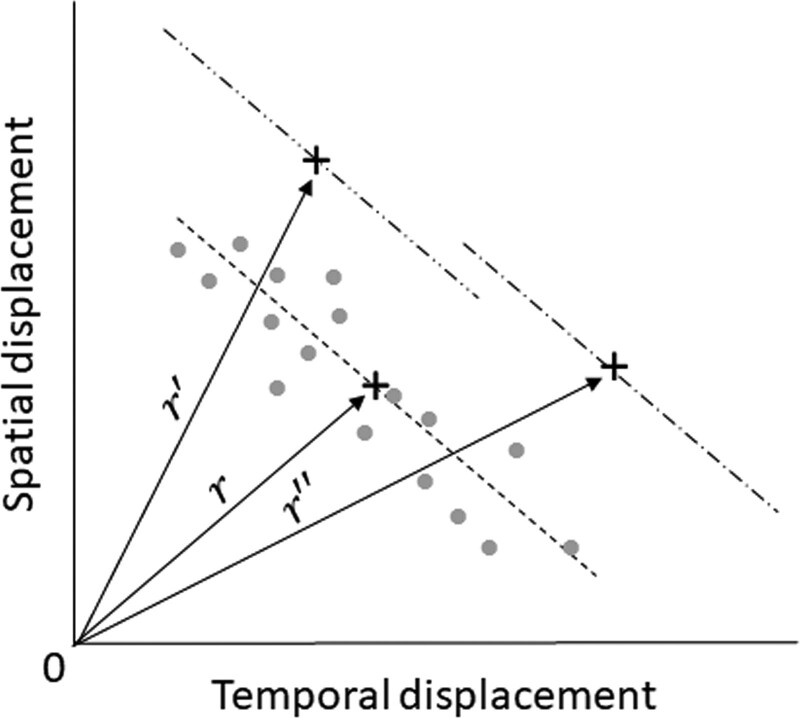
Hypothesized alterations in the distance are based on the spatial and temporal displacement relationship. The processing ability, reliant on the spatial and temporal displacement relationship, is expected to shift upwards, and the distance should extend from r to $r^{\prime }$ as spatial displacement augments. Conversely, with an increase in temporal displacement, the processing ability based on spatial and temporal displacement relationship should experience a rightward shift, and the distance should similarly extend from r to $r^{\prime \prime }$.


For each participant, we averaged the spatial displacement (cm) between the touch point and the target center point, and the temporal displacement (sec) between the touch and target sounds for Test A and B, respectively. Additionally, to estimate the processing ability based on the trade-off between accuracy and speed, we measured the distance (r) for each participant between the ultimate processing ability (i.e., when both spatial and temporal displacements are zero) and the current processing ability, considering the relationship between spatial and temporal displacement (see Fig. 2). Thus, the distance (i.e., processing ability), based on the relationship of spatial and temporal displacement, was estimated using the following equations:

$$\begin{array}{l} Spdis=\alpha +\beta \;\frac{\sum Tedis}{n} \end{array}$$ (1)

$$\begin{array}{l} Distance=\sqrt{{\left(\alpha +\beta \;\frac{\sum Tedis}{n}\right)}^{2}+{\left(\frac{\sum Tedis}{n}\right)}^{2}} \end{array}$$ (2)

where α is the y-intercept of the spatial displacement, representing a scenario where temporal displacement is zero; β is the slope of the relationship between spatial and temporal displacement, reflecting individual-specific changes in this relationship; Spdis and Tedis represent spatial and temporal displacement, respectively; and n is the number of data points collected during Test A or B of EHCPPT. We hypothesized that an increase in spatial displacement would result in an upward shift in the processing ability based on the relationship between spatial and temporal displacement, and consequently, the distance would also lengthen. Conversely, if the temporal displacement increases, it should cause the processing ability based on the relationship between spatial and temporal displacement to shift rightward, and the distance should lengthen as well.

### 2.2. Participants

The sample size was based on a desired statistical power of 80% for the detection of changes in spatial and temporal displacements, with an effect size of 0.10 and a two-sided α-level of 0.05. Utilizing these parameters in G*Power 3^[12]^ indicated a required sample size of 127. Subsequently, we enlisted 130 older participants. The eligibility criteria encompassed individuals aged 60 years and older, who had motor and cognitive functions assessable per the testing protocol. The average age of participants meeting the eligibility was 80.4 ± 7.4 years. The sample consisted of 78 women and 52 men; it included 53 healthy participants, 28 individuals with a history of stroke, 15 patients with fractures, 5 with Parkinson’s disease, 8 with spinal cord stenosis, and 21 with other conditions.

### 2.3. Assessments of reproducibility

Reproducibility was evaluated by administering the EHCPPT across two separate trials, the first and second half. The intraclass correlation coefficient (ICC) and Bland-Altman plots were utilized to assess reproducibility. The ICC was used for variance estimation.^[13]^ Bland-Altman analysis is a method used to examine the agreement between two different measurement techniques.^[14]^ Plotting the difference against the mean allows for the exploration of any potential relationship between measurement error and the true value. Specifically, if 95% of the values are within 2 standard deviations (SD) of this mean difference, then the 2 testing modalities may be considered interchangeable.

### 2.4. Assessments of validity

The EHCPPT was developed to evaluate rhythmic movements, accommodating various levels of difficulty, including the equal rhythm of Test A and the random rhythm of Test B. We hypothesized that if both spatial and temporal displacements increase in Test B, then the distance, reflecting the disparity between ultimate and current processing abilities based on the relationship between spatial and temporal displacements for Test B, should be greater than that for Test A. Consequently, to assess construct validity, the distances for Test A and Test B were compared using a paired *t* test.

Moreover, the EHCPPT was devised to evaluate rhythmic movements in a manner that gauges the relationship between spatial and temporal displacement, reflecting diverse motor and cognitive dysfunctions in older individuals and patients with various impairments. Aging is typically characterized by a general decline in muscle mass.^[15,16]^ Research on older community-dwelling populations indicates that grip strength can forecast declines in overall motor function and increased mortality risk.^[17,18]^ Hence, grip strength could serve as a significant predictor of motor function in the older individuals.^[19]^ Furthermore, the Trail Making Test (TMT), consisting of parts A and B, is a prevalent cognitive measure assessing functions like attention, visual search and scanning, sequencing, and shifting, and is thought to evaluate different cognitive processes.^[20,21]^ The part A of the TMT (TMT-A) is believed to be more associated with attention, whereas TMT-B is linked to executive function.^[20]^ However, age correlations were more pronounced in TMT-A than in TMT-B, as age-related individual variances in cognitive processes were less in TMT-B compared to TMT-A.^[21]^

We hypothesized that if the EHCPPT accurately mirrors various motor and cognitive dysfunctions in older individuals and patients with different impairments, then the distance, representing the discrepancy between ultimate and current processing ability based on the relationship of spatial and temporal displacement, should exhibit correlation with grip strength and the score of TMT-A. Consequently, to evaluate concurrent validity, we determined whether grip strength and the score of TMT-A were correlated to the aforementioned distance in the EHCPPT, employing the Pearson correlation coefficient.

This study was conducted after obtaining the consent of all the subjects and approval from the following ethics review committees: the Tokyo Kasei University Ethics Review Committee (approval number SKE2021-22). The protocol of the research project conforms to the provisions of the Declaration of Helsinki (as revised in Brazil 2013).

## 3. Results

### 3.1. Reproducibility

For the Test A of the EHCPPT, the mean ± SD of spatial and temporal displacements were 0.184 ± 0.149 and 0.044 ± 0.228, respectively. Similarly, for the Test B of the EHCPPT, they were 0.220 ± 0.213 and 0.606 ± 0.149, respectively.

**Figure 3.**
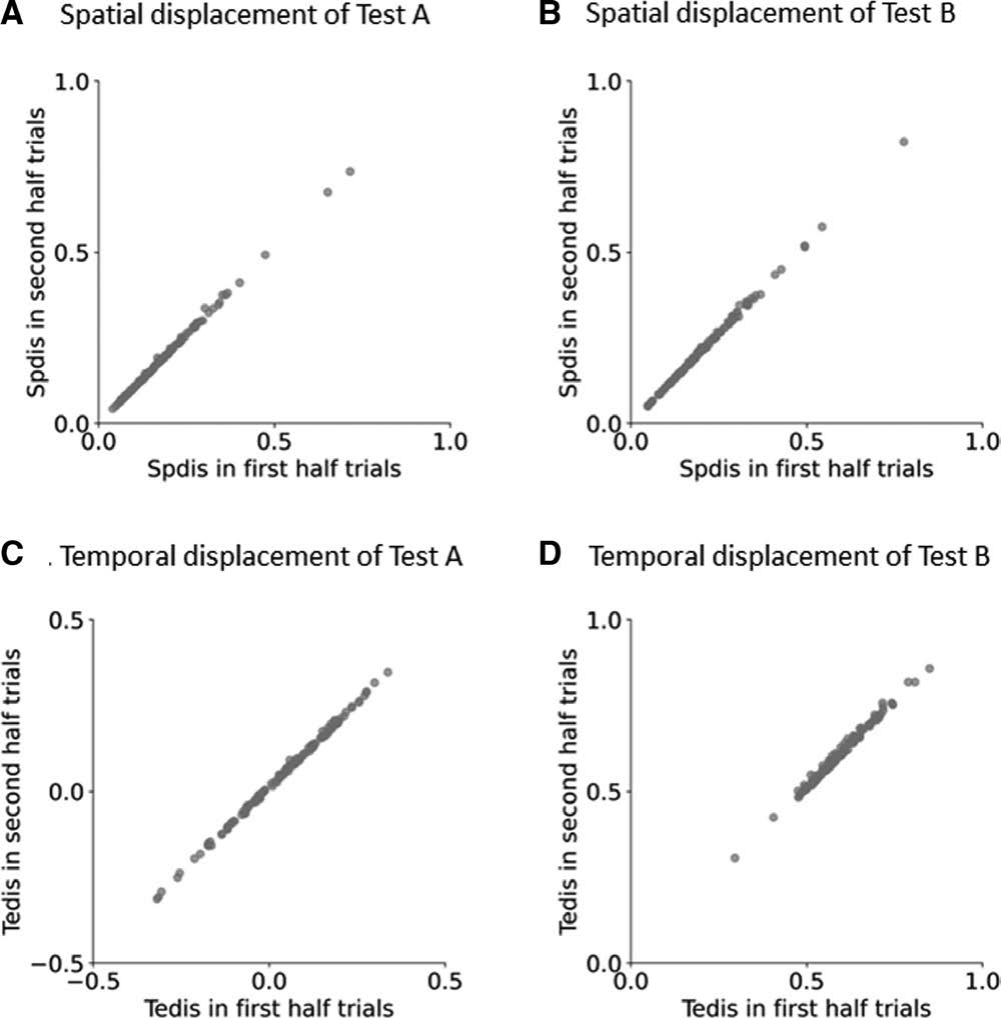
The scatterplots depict spatial (A) and temporal (C) displacements from the first and second half measurements for Test A of the EHCPPT. The ICCs for spatial and temporal displacements from Test A were 0.996 (*P* < .0001) and 0.995 (*P* < .0001), respectively. Scatterplots also represent spatial (B) and temporal (D) displacements from the first and second half measurements for Test B of the EHCPPT. The ICCs between spatial and temporal displacements from Test B were 0.992 (*P* < .0001) and 0.980 (*P* < .0001), respectively. EHCPPT = Eye–Hand Coordination Pointing with Pencil Test, ICCs = intraclass correlation coefficients, Spdis = spatial displacement, Tedis = temporal displacement.

Figure 3 presents the results of the first and second half measurements from Tests A and B of the EHCPPT. For Test A of the EHCPPT, the ICCs for spatial and temporal displacements were 0.996 (*P* < .0001) (Fig. 3A) and 0.995 (*P* < .0001) (Fig. 3C), respectively. Similarly, for Test B of the EHCPPT, the ICCs were 0.992 (*P* < .0001) (Fig. 3B) and 0.980 (*P* < .0001) (Fig. 3D), respectively.

**Figure 4.**
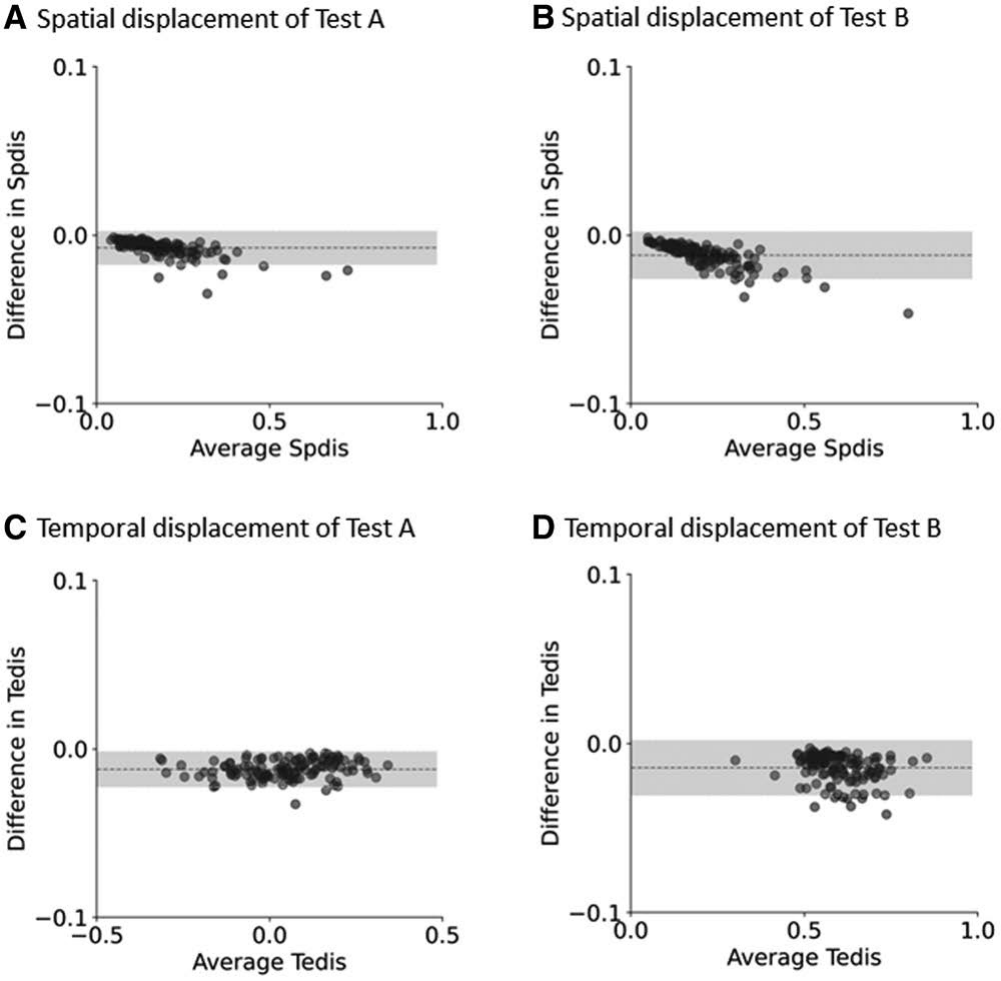
Bland-Altman plots illustrating the differences relative to the means of the first and second half measurements for spatial (A) and temporal (C) displacements in Test A of the EHCPPT. Dashed lines represent the average difference between the 2 measurements, and the shaded areas highlight the region within 2 SDs of this mean difference. For Test A, 95% of difference values for spatial displacement and 98% for temporal displacement fell within 2 SDs of the mean difference. The figure also depicts Bland-Altman plots for spatial (B) and temporal (D) displacements in Test B of the EHCPPT, wherein 96% and 95% of difference values for spatial and temporal displacements, respectively, were within 2 SDs of the mean. EHCPPT = Eye–Hand Coordination Pointing with Pencil Test, SD = standard deviation, Spdis = spatial displacement, Tedis = temporal displacement.

Bland-Altman plot visually represents the difference in the mean of spatial and temporal displacements between the first and second half measurements in Tests A and B (Fig. 4). In Test A, 95% of the difference values in spatial displacement (Fig. 4A) and 98% in temporal displacement (Fig. 4C) fell within 2 SD of the mean. Similarly, in Test B, 96% of the difference values in spatial displacement (Fig. 4B) and 95% in temporal displacement (Fig. 4D) were within 2 SD of the mean.

### 3.2. Validity

**Figure 5.**
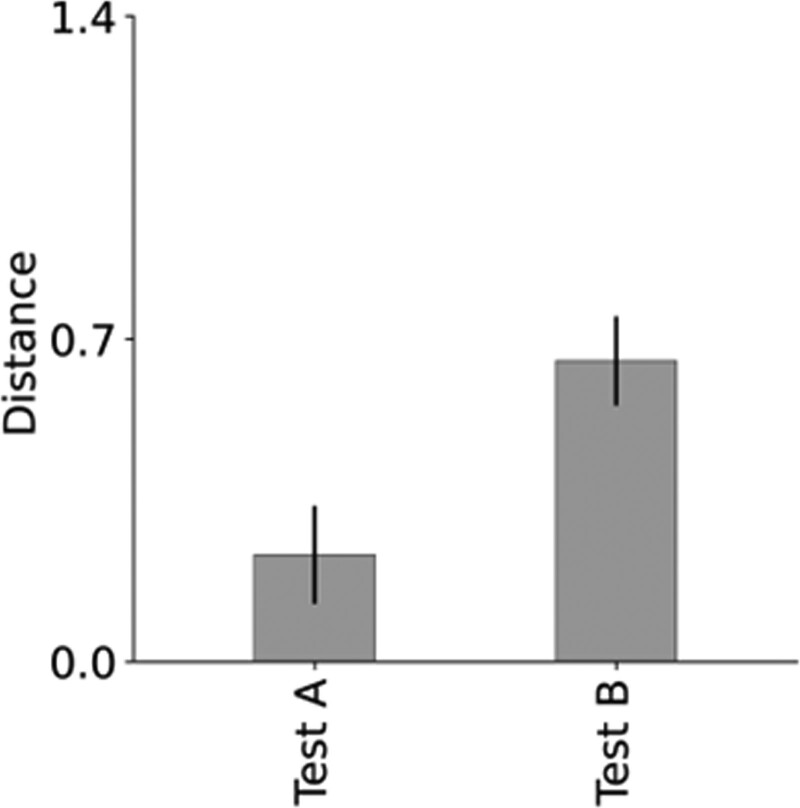
Bar chart illustrating the difference in the distance of the EHCPPT between Test A and B. The distance between ultimate and current processing abilities, based on the relationship of spatial and temporal displacements, was significantly shorter in Test A compared to Test B. EHCPPT = Eye–Hand Coordination Pointing with Pencil Test.

Figure 5 presents a bar chart depicting the difference in distance in the EHCPPT between Tests A and B to assess construct validity. The distance between ultimate and current processing ability, based on the relationship of spatial and temporal displacement, was significantly shorter in Test A compared to Test B.

**Figure 6.**
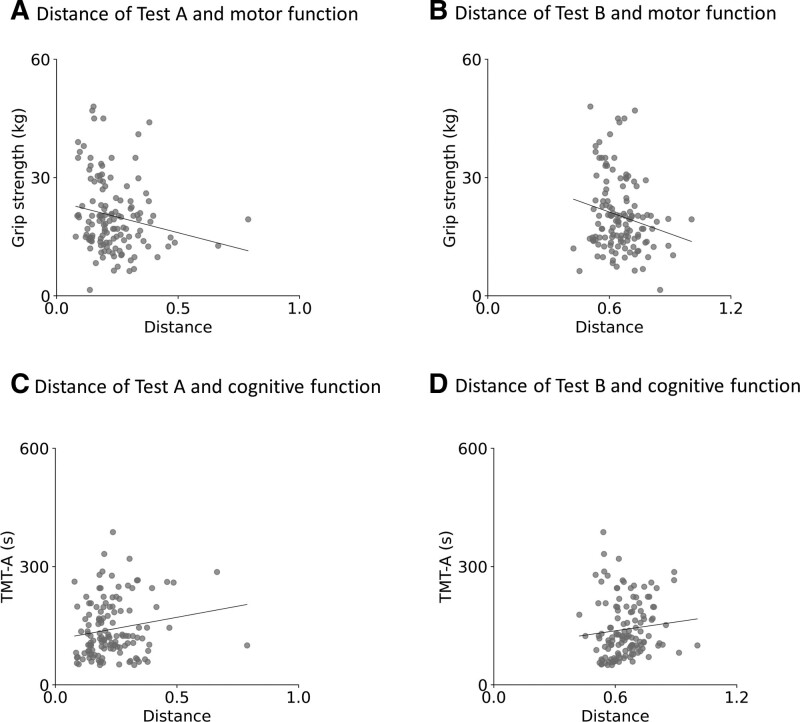
Scatter plots illustrating the Pearson correlation for the relationship between the distance and grip strength (A) and TMT-A scores (C) for Test A of EHCPPT. In Test A, there was a significant correlation between the distance and both grip strength and TMT-A scores. Additionally, scatter plots are shown depicting the Pearson correlation for the relationship between the distance and grip strength (B) and TMT-A scores (D) for Test B of EHCPPT. Similarly, in Test B, the distance was also significantly correlated with grip strength and TMT-A scores. EHCPPT = Eye–Hand Coordination Pointing with Pencil Test, TMT-A = Trial Making Test.

Figure 6 displays scatter plots representing the Pearson correlation coefficient to illustrate the relationship between distance and motor and cognitive functions, evaluating concurrent validity. In Test A, the distance demonstrated a significant correlation with both grip strength (Fig. 6A) and TMT-A scores (Fig. 6C). Similarly, in Test B, a significant correlation was observed between the distance and grip strength (Fig. 6B) as well as TMT-A scores (Fig. 6D).

## 4. Discussion

In this study, we have presented the reliability and validity of the EHPPT, a tool that assesses rhythmic movement function, based on the relationship between spatial and temporal information. The EHPPT demonstrated reproducibility as well as construct and concurrent validity.

Spatial and temporal information must be processed continuously during rhythmic movement. The ability to process this spatial and temporal information is a fundamental property of various rhythmic movements encountered in daily life.^[2–4]^ Consequently, we devised a new measure, EHCPPT, to assess the spatial and temporal displacement inherent in rhythmic movement. The repeatability of spatial and temporal displacements exhibited high consistency in both Tests A and B of EHCPPT. Previously, studies have investigated the visual and auditory memories in younger and older adults and suggested that preservation of ability for visual than auditory memory was better in older than younger adults.^[22]^ Additionally, studies compared visual and auditory temporal integration in younger and older adults, and noted that temporal integration time for older participants was more than for younger adults.^[23]^ In our study, temporal displacement of the EHCPPT was related to the processing of auditory information to motor output because participants were required to tapping on hearing a beep sound. However, spatial displacement was related to the processing visual information to motor output because participants were required to tap on the target (i.e., the center of the concentric circle). Hence, under conditions of heightened auditory memory, the temporal displacement of EHCPPT might exhibit greater magnitude and lower consistency compared to spatial displacement. Nevertheless, our EHCPPT demonstrated high reproducibility for both spatial and temporal displacements. These results suggest that both types of displacements in the EHCPPT are stable and valuable for assessing the rhythmic movement function, grounded in the relationship between spatial and temporal information. Moreover, this stable measurement of spatial and temporal displacement with EHCPPT can facilitate the accurate estimation of each participant’s distance between ultimate and current processing ability, based on the relationship between spatial and temporal displacements.

For construct validity, the distance between the ultimate and current processing ability, based on the relationship between spatial and temporal displacements, was significantly lower in Test A compared to Test B. These findings suggest that the distance measure in EHCPPT can effectively assess rhythmic movements, reflecting the varying difficulties presented in Tests A and B. Furthermore, regarding concurrent validity, this distance was significantly correlated with grip strength and TMT-A scores. These findings suggest that the distance measured by EHCPPT may vary in accordance with the severity of motor and cognitive impairments. Previous studies on rhythmic movement tasks attempted to identify the characteristics of rhythmic movement disorders in older adults – specifically, patients with Parkinson’s and cerebrovascular diseases – using a three-dimensional motion capture analysis.^[24]^ However, the clinical applicability of traditional rhythmic movement tasks is constrained due to their inability to assess processing ability centered on spatial and temporal displacement and the necessity for precise calibration of large, costly measurement devices. In contrast, the EHCPPT can evaluate processing ability predicated on spatial and temporal displacements and can be administered while the participant is seated, in less time, inducing minimal fatigue. Hence, simple and objective evaluations employing the distance measure between ultimate and current processing ability, grounded on the spatial and temporal displacement relationship in EHCPPT, could facilitate the preliminary assessment of motor and cognitive functions in the elderly.

Preliminary reports reported that the EHCPPT could assess symptoms of Ataxia and Parkinson’s disease.^[11]^ In the present study, the distance between ultimate and current processing ability, based on the relationship of spatial and temporal displacement, was correlated to motor and cognitive functions. These findings suggest that this distance can serve as an evaluative indicator in rhythmic coordination tasks, reflecting the motor and cognitive capabilities of older individuals. Subsequent research should explore whether alterations in this distance can depict changes in motor and cognitive dysfunctions in older individuals post rhythmic movement training.

### 4.1. Limitation

Our study has 3 main limitations. Firstly, our assessment was solely focused on spatial and temporal displacement during rhythmic movement. Future studies will need to employ waveform analysis to quantitatively assess the rhythmic motions of older individuals with varying impairments. This is crucial to corroborate EHCPPT against other quantitative metrics of reaction time, finger tapping, and the balance between speed and accuracy.

Secondly, a significant challenge with administering tests via computerized devices to older individuals with cognitive disorders is their inability to operate these devices. This unfamiliarity can also lead to performance anxiety.^[25]^ Therefore, based on our findings, further research is needed to create a user-friendly protocol for older individuals with cognitive disorders to comfortably use computerized devices like EHCPPT. Such a protocol would enhance the generalizability of EHCPPT.

Thirdly, tests using computerized devices aren’t practical for clinical scenarios without such equipment. Consequently, there’s a pressing need for further research to design devices that are portable, time-efficient, and cost-effective.

## 5. Conclusion

The EHCPPT demonstrates excellent reproducibility in spatial and temporal displacements, and the distance between ultimate and current processing abilities, based on the spatial and temporal displacement relationship, exhibits construct and concurrent validity in older populations. The EHCPPT may function as a measure of rhythmic movement, reflecting both motor and cognitive functions. Further studies are essential to explore the relationship between the EHCPPT’s displacement-based distance and the effects of intervention. Additionally, future research should elucidate the EHCPPT’s responsiveness to clinical changes to establish its utility as a clinically valuable tool.

## Author contributions

**Conceptualization**: Kazuo Saito, Makoto Suzuki.

**Data curation**: Maki Koyama.

**Formal analysis**: Makoto Suzuki, Junichi Yamamoto, Toshiko Futaki.

**Funding acquisition**: Junichi Yamamoto, Toshiko Futaki.

**Investigation**: Kazuo Saito.

**Methodology**: Makoto Suzuki, Junichi Yamamoto.

**Project administration**: Toshiko Futaki.

**Resources**: Kazuo Saito.

**Software**: Maki Koyama.

**Supervision**: Junichi Yamamoto, Toshiko Futaki.

**Validation**: Makoto Suzuki.

**Visualization**: Makoto Suzuki.

**Writing – original draft**: Kazuo Saito, Makoto Suzuki.

**Writing – review & editing**: Kazuo Saito, Suzuki Makoto, Maki Koyama, Junichi Yamamoto, Toshiko Futaki.
